# Prevalence of road traffic accidents and associated factors among individuals aged 18 to 69 years in Rwanda. Analysis of STEP survey 2021–2022

**DOI:** 10.1186/s12889-026-27096-8

**Published:** 2026-03-23

**Authors:** Israel Cyubahiro Munyambaraga, Placide Shema Niyonshuti, Jean Luc Benimana, Pascal Mugemangango, Agathe Nyirambyeyi, Michael Habtu, Pasteur Dushimimana

**Affiliations:** 1 College of Medicine and Health Sciences, School of Public Health, University of Rwanda, Kigali, Rwanda; 2 College of Medicine and Health Sciences, School of medicine and pharmacy, University of Rwanda, Kigali, Rwanda; 3 Epi-Care Green Africa, Kigali, Rwanda; 4 University Teaching Hospital of Kigali (CHUK), Kigali, Rwanda; 5 Ministry of health, Kigali, Rwanda

**Keywords:** Factors, Road traffic accident, STEP survey and prevalence

## Abstract

**Background:**

Road traffic accidents (RTAs) are a major global public health problem, causing approximately 1.19 million deaths and 50 million injuries annually, with a disproportionate burden in low- and middle-income countries. In Rwanda, RTAs have increased in recent years, largely attributed to speeding, negligence, and alcohol use, despite ongoing prevention efforts.

**Objective:**

This study aimed to determine the prevalence of road traffic accidents and identify associated socio-demographic factors among adults in Rwanda.

**Methods:**

A cross-sectional analysis was conducted using secondary data from the 2021–2022 Rwanda WHO STEPwise Approach to Surveillance (STEPS) Survey. The study included 5,173 adults aged 18–69 years, representing a weighted population of 4,974,815. Road traffic accidents were assessed using self-reported information from participants and included accidents of any severity within the previous 12 months. Weighted descriptive statistics, bivariate analysis, and multivariable logistic regression were performed using Stata version 17. Adjusted odds ratios (AORs) with 95% confidence intervals (CIs) were estimated, with statistical significance set at *p* < 0.05.

**Results:**

The weighted prevalence of RTAs was 6.58% (95% CI: 5.54%–7.78%) within 12 months. Multivariable analysis identified key predictors of road traffic accidents. Females (AOR = 0.405, 95% CI: 0.277–0.593), residents of the Southern Province (AOR = 0.464, 95% CI: 0.227–0.947), married/cohabitating (AOR = 0.536, 95% CI: 0.328–0.875), divorced/separated/widowed (AOR = 0.353, 95% CI: 0.173–0.721), and those with primary (AOR = 0.637, 95% CI: 0.426–0.952) or secondary education (AOR = 0.466, 95% CI: 0.242–0.898) had lower odds, while middle-income status increased risk (AOR = 1.473, 95% CI: 1.018–2.132). A reversal in direction between COR and AOR was observed for Eastern Province and secondary education, with secondary education remaining significantly protective.

**Conclusion:**

Road traffic accidents remain a significant public health concern in Rwanda. Socio-demographic factors such as sex, Southern Province, marital status, education level, income, and media exposure are important determinants of RTAs. These findings highlight the need for targeted, evidence-based road safety interventions and further research incorporating behavioral, environmental, and vehicle-related risk factors.

**Clinical Trial Registration:**

Not applicable.

## Background

Road traffic accidents(RTAs) are a major public health concern globally, resulting in about 1.19 million deaths each year and leaving around 50 million people sustain non-fatal injuries [1–3]. Survivors often suffer from lasting impairments and disabilities that negatively impact their overall quality of life [4]. Approximately 3,700 people die each day due to road traffic accidents, with nearly half of these deaths occurring among vulnerable road users, including pedestrians, cyclists, and motorcyclists [3]. The burden of road traffic injuries (RTIs) remains a major global public health challenge and is projected to become the seventh leading cause of death worldwide by 2030 [1]. In 2019, low- and middle-income countries accounted for approximately 92% of all road traffic–related deaths, despite these settings having a disproportionately small share of the global motor vehicle fleet [3]. Moreover, the risk of dying from a road traffic crash in low-income countries is estimated to be about three times higher than in high-income countries, highlighting persistent global inequities in road safety and trauma care systems [3]. In addition to the health impact, road traffic injuries generate substantial economic losses, estimated at around USD 518 billion annually, corresponding to 1–3% of gross domestic product in many countries, with the greatest burden borne by low- and middle-income countries [3, 5, 6].

Africa bears the highest burden of road traffic accidents (RTAs), with a pooled overall prevalence of 38.8% among public transportation users [5]. Key risk factors include driving experience, alcohol use, and using mobile phones while driving [5], The highest prevalence was observed in Congo and Central Africa at around 68%, whereas Libya and North Africa reported the lowest, ranging from 16% to 25% [5].Prevalence of RTA in Juba, South Sudan and Kenya were 7.3%, 6.3% and 6.3% respectively [7, 8]. Household-based study in Ethiopia reported that 123 out of 75,271 household members experienced RTAs during the reference period [9]. Additionally, a systematic review of 310,660 trauma patients across sub-Saharan Africa reported a median RTA proportion of 32%, with a median mortality of 5% [10].

Rwanda has seen a steady increase in road traffic accidents and related deaths with high prevalence of disability of 36.14% [11, 12]. According to the National Institute of Statistics of Rwanda (NISR) and the Rwanda National Police(RNP), the number of road traffic accidents has fluctuated but demonstrated a general upward trend, Reported crashes increased sharply from 4,203 in 2020 to 8,639 in 2021, 10,334 in 2022, and 9,995 in 2023 [13, 14]. Despite this rise, fatal accidents remained relatively stable, ranging from 597 in 2018 to 761 in 2023, while the national death rate due to RTIs varied modestly between 4.7 and 5.7 per 100,000 population from 2019 to 2022 suggesting improved emergency response and post-crash care [13–15]. RTAs in Rwanda are mainly caused by negligence, speeding, poor maneuvering, and alcohol use, with motorcyclists, passengers, and pedestrians being the most affected [15]. The nationwide introduction of ASE cameras in Rwanda in April 2021 has contributed to a reduction in road traffic mortality, resulting in a 38.16% decrease in monthly deaths compared to the period prior to their installation [16]. The implementation of the Gerayo Amahoro campaign has contributed to a reduction in traffic-related deaths in Rwanda; however, road traffic accidents continue to increase despite the measures taken [15].

Conceptually, road traffic accidents arise from the interaction of individual socio-demographic characteristics, behavioral practices, and the broader environmental and policy context [17]. Factors such as age, sex, residence, education, and socioeconomic status influence exposure to traffic and risk-taking behaviors, while alcohol consumption, speeding, mobile phone use, and media exposure shape driving practices and risk perception [18]. These individual-level determinants operate within structural conditions, including urbanization and enforcement intensity, which further modify RTA risk [19]. In Rwanda, monitoring of RTAs relies largely on police and health facility surveillance systems that primarily capture fatal or severe cases and provide limited insight into upstream, population-level determinants and non-fatal events.

Despite the implementation of the Gerayo Amahoro campaign and the nationwide rollout of Automated Speed Enforcement (ASE) cameras which have contributed to reductions in road traffic related mortality the overall number of RTAs in Rwanda continues to increase [15, 16]. Evidence on the population level determinants of RTAs in the post-intervention period remains limited. Although the WHO STEPwise Approach to Surveillance (STEPS) Survey was originally designed for non-communicable disease risk factor monitoring, it offers nationally representative, community-based data on injuries, socio-demographic characteristics, behavioral factors, and media exposure relevant to road traffic injury risk [20]. By analyzing data from the 2021–2022 Rwanda STEPS Survey, this study addresses a critical evidence gap by identifying population groups at higher risk of RTAs and testing whether socio-demographic factors remain significant predictors despite strengthened enforcement measures, thereby informing targeted and prevention-oriented road safety interventions.

## Methods

### Research design

This study used a cross-sectional design based on secondary analysis of data from the 2021–2022 Rwanda WHO STEPwise Approach to Surveillance (STEPS) Survey, a nationally representative population-based survey conducted by the Rwanda Biomedical Center. The study aimed to identify factors associated with road traffic accident among Rwandan aged 18 to 69 years.

### Study setting

The 2021–2022 Rwanda STEPS Survey was conducted nationwide using the WHO STEPwise approach to NCD risk factor surveillance. The survey employed an adapted WHO STEPS questionnaire and was implemented in three sequential components. STEP 1 collected behavioral and injury-related information through face-to-face interviews, including self-reported involvement in road traffic accidents. STEP 2 obtained physical measurements such as blood pressure, height, weight, and body mass index (BMI), while STEP 3 included biochemical measurements, such as fasting blood glucose and lipid profiles.

Although STEPS is not a dedicated road traffic injury surveillance system, its standardized methodology and nationally representative sampling framework allow for the assessment of population-level patterns of self-reported RTAs, including non-fatal events that may not be captured in police or health facility based data. However, the use of STEPS data also imposes important limitations, including reliance on self-reported RTA history, lack of detailed information on crash circumstances (e.g., type of road user, injury severity, vehicle characteristics), and the inability to establish temporal or causal relationships due to the cross-sectional design. These limitations were considered in the interpretation of the findings.

### Study population

The study included female and male aged 18 to 69 years who participated in the 2021–2022 STEP Survey [21]. Exclusions were made for those with mental disabilities, cognitive impairments, or hearing and speech challenges that hindered oral survey participation. While the survey acknowledged that individuals with disabilities face similar non-communicable disease (NCD) risks as the general population, it was not designed to generate statistically stable estimates for this subgroup. The target population consisted of female and male within the specified age range, excluding those unable to participate in oral interviews due to disabilities. Individuals under 18 were excluded because the STEPS survey focuses on adult populations.

### Sample size

This study used total enumeration of all eligible respondents included in the 2021–2022 Rwanda WHO STEPwise Approach to Surveillance (STEPS) Survey. No formal sample size calculation was performed because the analysis relied on secondary data. The STEPS Survey initially interviewed 5,776 unweighted adults aged 18–69 years. After excluding records with missing information on the outcome variable, the final analytical (unweighted) sample consisted of 5,173 respondents. To ensure national representativeness and account for the complex survey design, sampling weights were applied; consequently, the weighted estimates represent an adult population of approximately 4,974,815 individuals in Rwanda.

Given the use of total enumeration and the observed prevalence of road traffic accidents, the effective sample size and number of outcome events were considered adequate for multivariable logistic regression analysis, allowing for stable estimation of adjusted odds ratios.

### Sampling technique

The Rwanda STEPS Survey employed a multistage cluster sampling design to achieve national representativeness. In the first stage, 400 enumeration areas (clusters) were selected using probability proportional to size (PPS) from a national sampling frame of 14,837 urban and rural villages provided by the National Institute of Statistics of Rwanda (NISR), comprising 120 urban and 280 rural clusters. In the second stage, 15 households were systematically selected from each cluster. In the third stage, one eligible adult (18–69 years) was randomly selected from each household, yielding a total unweighted sample of 5,776 respondents.

### Data collection

Data were obtained from the 2021–2022 Rwanda WHO STEPwise Approach to Surveillance (STEPS) Survey, conducted by the Rwanda Biomedical Center in collaboration with the World Health Organization. The STEPS methodology uses standardized questionnaires, physical measurements, and biochemical assessments to collect population-level health data. For this secondary analysis, variables relevant to road traffic accidents and their potential determinants were extracted from the cleaned STEPS dataset using a predefined data extraction framework guided by existing literature and study objectives.

### Study variables

#### Outcome variable

The outcome variable was self-reported involvement in a road traffic accident (RTA) during the 12 months preceding the survey, as assessed in the WHO STEPS survey. Respondents reported whether they had experienced any RTA, regardless of injury severity, road user role, or whether the event was reported to police or health facilities. Responses were coded as a binary variable (Yes/No). The STEPS survey does not capture accident severity, frequency, exposure time, or role in the crash; therefore, individuals reporting one or multiple accidents were classified similarly. Accordingly, the outcome represents overall RTA occurrence at the community level and should be interpreted as an indicator of self-reported accident involvement rather than crash severity or causality.

#### Explanatory variables

Explanatory variables included socio-demographic, behavioral, and contextual factors selected based on theoretical relevance and prior evidence. Socio-demographic variables comprised province of residence (East, Kigali City, North, South, and West), place of residence (urban/rural), sex, age group (18–29, 30–44, 45–59, and 60–69 years), education level, marital status, wealth index, and employment status. Behavioral variables included seatbelt use, helmet use, alcohol consumption, and smoking status. Media exposure was defined using the STEPS indicator of regular access to mass media (radio, television, or newspapers) and was included as a proxy for broader socioeconomic position, urbanization, and lifestyle characteristics rather than media use while driving.

### Statistical analysis

Data analysis was conducted using Stata version 17. Given that the data were derived from the nationally representative 2021–2022 Rwanda STEPS Survey, sampling weights were applied using the svy command to account for the complex survey design and ensure nationally representative estimates. Weighted descriptive statistics, including frequencies and percentages, were used to summarize participants’ characteristics. Bivariate analysis was performed to assess the association between each independent variable and the outcome variable, with variables showing a p-value < 0.05 considered statistically significant and included in the multivariable logistic regression model to identify independent predictors of road traffic accidents. Multicollinearity was assessed using the Variance Inflation Factor (VIF), and variables with VIF values greater than 10 were excluded from the final model to maintain model stability and reliability. Model fit was assessed using information criteria, including the Akaike Information Criterion (AIC) and the Bayesian Information Criterion (BIC).

### Ethical consideration

The Rwanda National Ethics Committee granted ethical clearance for the primary survey. The Rwanda Biomedical Centre approved the use of the dataset for secondary analysis. This study obtained ethical clearance under reference number CMHS/IRB/401/2025 from the CMHS Institutional Review Board (IRB).

## Result

### Prevalence of road traffic accident

**Fig. 1:**
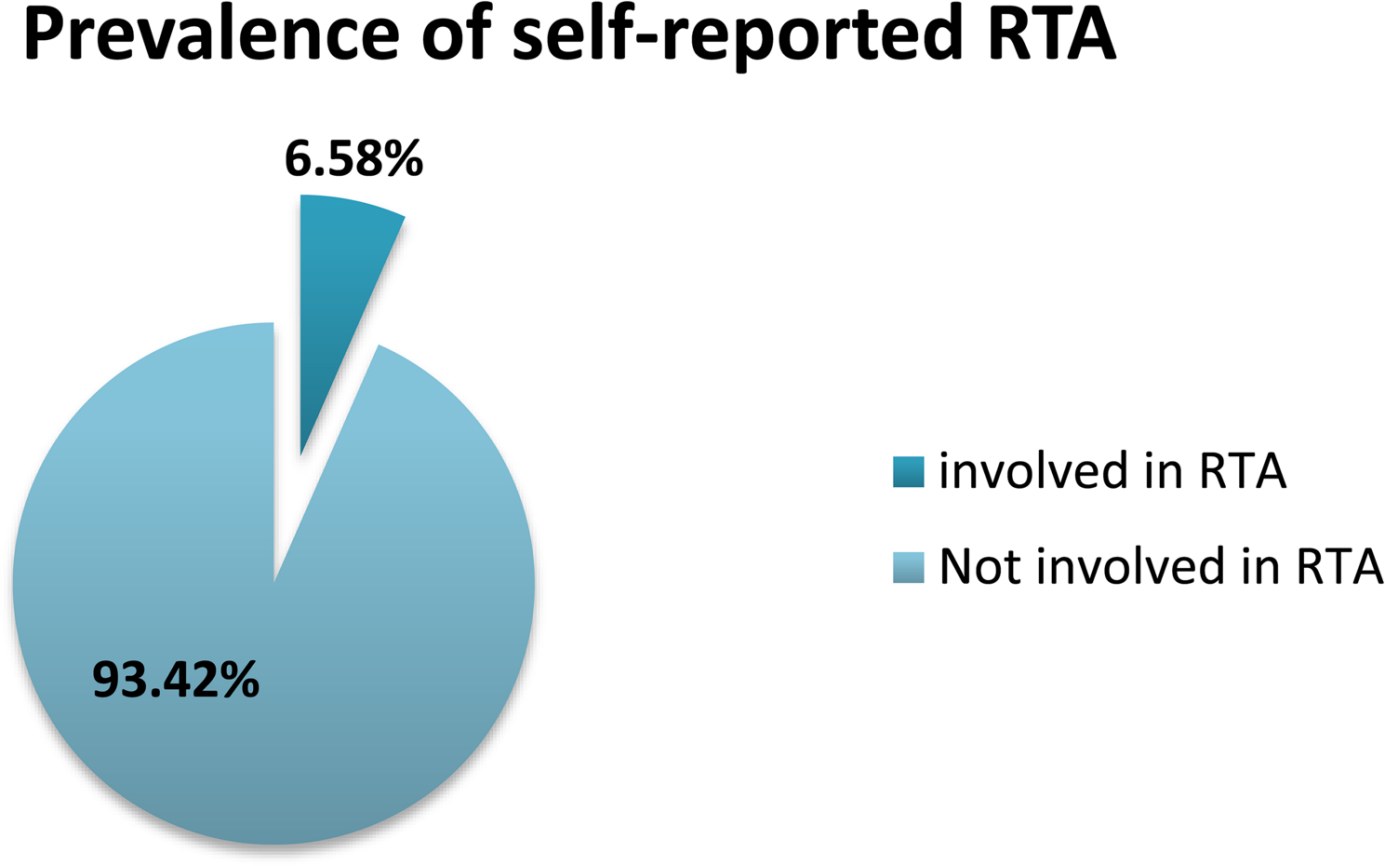
Weighted prevalence of RTA

**Fig. 2:**
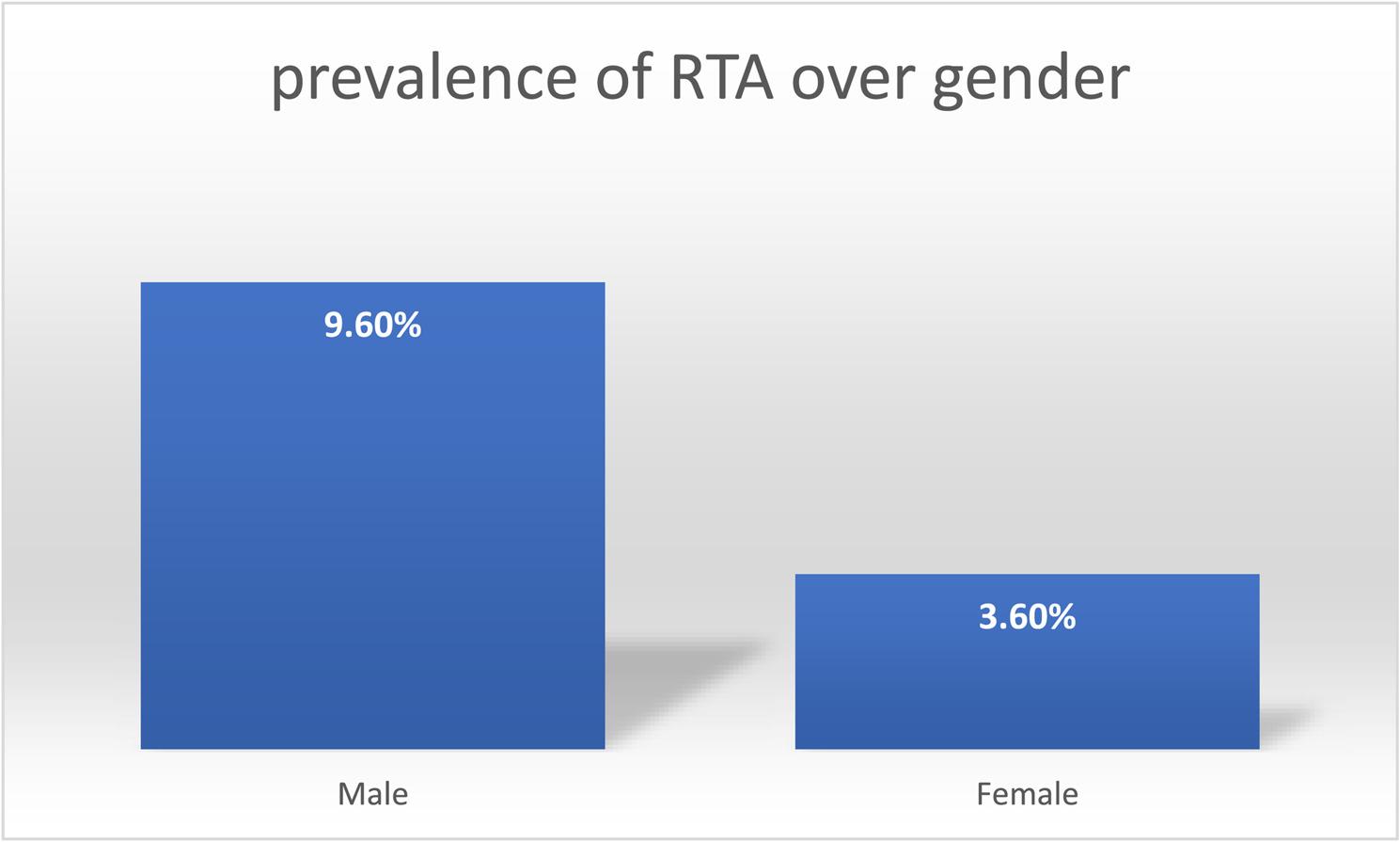
Weighted prevalence of RTA over gender

Among the weighted adult population of 4,974,815 individuals in Rwanda, the prevalence of self-reported road traffic accidents regardless severity was 6.58% (95% CI: 5.54%–7.78%) within 12 months (Fig. 1).When disaggregated by sex, the prevalence of RTAs was notably higher among men, at 9.60% (95% CI: 7.71%–11.82%), compared with women, who had a prevalence of 3.60% (95% CI: 2.71%–4.71%) (Fig. 2).

### Socio-demographic distribution of respondent

The study involved 4,974,815 weighted respondents, with an almost equal gender distribution. Most participants resided in rural areas (82.2%) and were from the Western (23.2%) and Eastern (22.6%) provinces. The majorities were aged 18–29 years (44.4%) and had attained primary education (59.4%), while only 2.2% had higher education. Nearly half (49.4%) belonged to the rich economic category, and about two-thirds (64.7%) were self-employed. Most respondents reported consuming alcohol (77.5%) and were non-smokers (93.2%).The use of safety measures was low, with only 21% always wearing seatbelts and 69.9% consistently using helmets. Majorities were married or cohabiting (59.8%) and most accidents occurred on roads or highways (20.9%), with limited media exposure reported by 13.7% of respondents (Table 1).

**Table 1:** weighted socio-demographic distribution of respondent

Variable	*N* (%)
Province	
East	1,123,904(22.6)
Kigali	715,207(14.3)
North	869,835(17.5)
South	1,112,209(22.4)
West	1,153,660(23.2)
Type of residence	
Rural	4,087,609(82.2)
Urban	887,206(17.8)
Gender	
Male	2,487,755(50.0)
Female	2,487,060(50.0)
Age group	
18–29	2,207,446(44.4)
30–44	1,593,800(32.0)
45–59	856,800(17.2)
60–69	316,769(6.4)
Education level	
No education	1,145,018(22.9)
Primary	2,954,080(59.4)
Secondary	768,693(15.5)
High education	107,024(2.2)
Economic status	
Poor	460,123(9.2)
Middle	2,058,387(41.4)
Rich	2,456,305(49.4)
Employment status	
Employee	770,532(15.5)
Self employed	3,219,195(64.7)
Unemployed	985,088(19.8)
Seatbelt	
All of the time	444,762(21.0)
Sometimes	380,260(18.0)
Never	1,292,734(61.0)
Helmet	
All of the time	1,838,431(69.9)
Sometimes	276,782(10.5)
Never	515,993(19.6)
Marital status	
Single	1,529,331(30.8)
Married/cohabitating	2,975,037(59.8)
Divorced/separated/widowed	468,781(9.4)
Consume alcohol	
Yes	3,853,992(77.5)
No	1,120,823(22.5)
Place of accident	
Road/street/highway	106,264(20.9)
Other	403,279(79.1)
Media	
No	4,273,086(86.3)
Yes	679,224(13.7)
Smoking status	
No	4,634,851(93.2)
Yes	339,964(6.8)

N:weighted frequency %:weighted percentage

### Multivariable logistic regression analysis of factors associated with RTA

Multivariable analysis identified key independent predictors of road traffic accidents. Females had significantly lower odds than males (AOR = 0.405, 95% CI: 0.277–0.593, *p* < 0.001). Residents of the South province were less likely to experience RTAs (AOR = 0.464, 95% CI: 0.227–0.947, *p* < 0.05). Married/cohabitating (AOR = 0.536, 95% CI: 0.328–0.875) and divorced/separated/widowed individuals (AOR = 0.353, 95% CI: 0.173–0.721) had lower risk compared to singles. Education was protective at primary (AOR = 0.637, 95% CI: 0.426–0.952) and secondary levels (AOR = 0.466, 95% CI: 0.242–0.898), while middle-income status increased risk (AOR = 1.473, 95% CI: 1.018–2.132). Media exposure showed a borderline association (AOR = 1.517, 95% CI: 0.972–2.368).A reversal in direction was observed between the crude odds ratio (COR) and adjusted odds ratio (AOR) for residents of the Eastern Province and for individuals with secondary education. However, the association for Eastern Province was not statistically significant after adjustment, while secondary education became significantly protective in the adjusted model. These findings are summarized in (Table 2).

**Table 2:** weighted bivariate and multivariable logistic regression analysis of factors associated with RTA

Variables	Road Traffic Accident	Bivariate logistic regression	multivariable logistic regression
	*N* (%)	COR, 95%CI	AOR,95%CI
Province			
East	98,753(8.8)	0.753[0.438–1.295]	1.044[0.547–1.995]
Kigali (Reference)	81,070(11.3)	1	1
North	46,832(5.4)	0.4451[0.234–0.846]*	0.614[0.286–1.315]
South	48,831(4.4)	0.359[0.201–0.642]***	0.464[0.227–0.947]*
West	51,619(4.5)	0.366[0.193–0.693]**	0.506[0.237–1.082]
Type of residence			
Rural (Reference)	244,149(6.0)	1	
Urban	82,956(9.4)	1.623[0.991–2.661]	
Gender			
Male (Reference)	238,091(9.6)	1	1
Female	89,014(3.6)	0.351[0.239–0.513]***	0.405[0.277–0.593]***
Age group			
18–29 (Reference)	186,597(8.5)	1	1
30–44	91,339(5.7)	0.658[0.481–0.901]	0.943[0.606–1.466]
45–59	39,734(4.6)	0.526[0.327–0.846]	0.838[0.475–1.477]
60–69	9435(3.0)	0.332[0.174–0.634]**	0.541[0.256–1.141]
Marital status			
Single (Reference)	161,736(10.6)	1	1
Married/cohabitating	154,341(5.2)	0.462[0.333–0.643]***	0.536[0.328–0.875]*
Divorced/separated/widowed	10,955(2.3)	0.202[0.118–0.344]***	0.353[0.173–0.721]**
Education level			
No education (Reference)	74,250(6.5)	1	
Primary	178,316(6.0)	0.926[0.629–1.363]	0.637[0.426–0.952]*
Secondary	53,779(7.0)	1.083[0.626–1.878]	0.466[0.242–0.898]*
High education	20,760(19.4)	3.471[1.273–9.455]*	1.386[0.47–4.087]
Economic status			
Poor	43,246(9.4)	1.995[1.183–3.364]*	1.398[0.786–2.487]
Middle	162,464(7.9)	1.648[1.165–2.331]**	1.473[1.018–2.132]*
Rich (Reference)	121,395(4.9)	1	1
Employment status			
Employee (Reference)	71,469(9.3)	1	1
Self employed	167,262(5.2)	0.536[0.338–0.849]**	0.681[0.426–1.089]
Unemployed	88,374(9.0)	0.963[0.52–1.786]	0.824[0.43–1.578]
Use seatbelt			
All of the time (Reference)	47,615(10.7)	1	
Sometimes	35,518(9.3)	0.859[0.354–2.085]	
Never	82,425(6.4)	0.568[0.301–1.073]	
Use helmet			
All of the time	160,473(8.7)	1.662[0.915–3.018]	
Sometimes	37,969(13.7)	0.602[0.336–1.077]	
Never (Reference)	28,103(5.4)	1	
Consume alcohol			
Yes (Reference)	247,724(6.4)	1	
No	79,381(7.1)	1.109[0.727–1.694]	
Place of accident			
Road/street/highway	10,868(10.2)	1.063[0.441–2.558]	
Other (Reference)	39,031(9.7)	1	
Media			
No (Reference)	243,798(5.7)	1	1
Yes	81,997(12.1)	2.269[1.512–3.406]**	1.517[0.972–2.368]*
Smoking status			
No (Reference)	313,003(6.8)	1	
Yes	14,102(4.1)	0.597[0.314–1.136]	

*AOR* Adjusted Odd Ratio, *COR* Crude Odd Ratio, *N*: weighted frequency, %: weighted Percentage *p*-value: *p < 0.05; **p < 0.01; P***<0.001

### Variance Inflation Factors (VIF) for independent variables included in the multivariable model

Multicollinearity among predictors was low, with VIF values ranging from 1.02 to 1.49 and a mean VIF of 1.16, well below the commonly used threshold of 10. The highest VIFs were observed for marital status (1.49) and age group (1.42), indicating minimal correlation between variables. These results confirm that the model estimates are stable and that the adjusted odds ratios are not biased by multicollinearity. (Table 3).

**Table 3:** Variance Inflation Factors (VIF) for Independent Variables Included in the Multivariable Model

Variable	VIF	1/VIF
Marital status	1.49	0.67
Age group	1.42	0.70
Education level	1.17	0.86
Economic status	1.07	0.93
Gender	1.07	0.93
Media exposure	1.05	0.95
Region	1.02	0.98
Employment status	1.02	0.98
Mean VIF	1.16	

### Model fit statistics for the multivariable logistic regression model

The multivariable model showed a meaningful improvement in fit compared with the null (intercept-only) model. Using 5,142 observations, the log-likelihood increased from − 984.83 in the null model to − 911.75 in the fitted model, indicating that the included predictors substantially enhanced the model’s explanatory capacity. The model estimated 19 parameters, and the resulting Akaike Information Criterion (AIC) of 1,861.49 and Bayesian Information Criterion (BIC) of 1,985.85 were relatively low, supporting the adequacy and parsimony of the specification. Taken together, the improvement in log-likelihood and the favorable information criteria justify retaining this model as an appropriate and well-fitting representation of the data for inference and interpretation.

## Discussion

Using weighted data from the 2021–2022 Rwanda STEP survey, this study estimated the prevalence of road traffic accidents (RTAs) among adults aged 18–69 years at 6.58% (95% CI: 5.54%–7.78%) within twelve month. This prevalence is lower than that reported in Ethiopia (32.8%), where the study population consisted of drivers a group inherently at higher risk of RTAs which may explain the observed difference [22]. The comparatively lower rate in Rwanda may reflect the effectiveness of ongoing national road safety initiatives, including stricter traffic law enforcement, improved road infrastructure, and continuous public education on safe road use. However, the prevalence remains slightly higher than that reported in Kenya (6.3%), which could be attributed to increasing vehicle ownership, rapid urbanization, and population mobility in Rwanda that elevate exposure to traffic risks [8].

This study found a substantially higher prevalence of road traffic accidents among men (9.60%, 95% CI: 7.71%–11.82%) compared with women (3.60%, 95% CI: 2.71%–4.71%) is consistent with evidence from Rwanda and other low- and middle-income countries. Similar sex disparities have been reported in studies from Sub-Saharan Africa and global analyses, which consistently show men bearing a disproportionate burden of RTAs [23, 24]. This pattern is commonly attributed to higher exposure to traffic among men, greater involvement in high-risk road use behaviors such as speeding and alcohol consumption, and occupational roles that require frequent travel or motorcycle use [24]. For instance, analyses of WHO Global Status Reports on Road Safety and studies from neighboring East African countries have documented RTA prevalence and injury rates among men that are two to four times higher than those among women, reinforcing the robustness of this finding across settings.

The contrast between crude and adjusted estimates in this study highlights the critical role of confounding in shaping observed associations. In unadjusted analyses, residents of the Eastern Province and individuals with secondary education appeared to have higher odds of road traffic accidents. After controlling for socio-demographic and contextual factors, the association for Eastern Province was no longer significant, while secondary education became significantly protective [25]. This reversal suggests that the apparent geographic risk was largely driven by differences in exposure, urbanization, and socioeconomic status rather than the province itself. The protective effect of secondary education aligns with previous studies showing that higher educational attainment enhances risk awareness, adherence to traffic rules, and safer road behaviors, reducing crash involvement [25–27]. These findings underscore the importance of multivariable modeling to account for confounding and to derive policy-relevant insights for targeted crash-prevention strategies in Rwanda.

Beyond prevalence, this study identified several factors associated with road traffic accidents. Notably, being female was significantly protective, with female being 0.42 times less likely to be involved in RTAs compared to men. This is consistent with a study in Kenya, where female household members were 0.72 less likely to be exposed to road traffic accidents compared to male household members [8]. Likewise, a study in Ethiopia reported that males had almost double the likelihood of being involved in road traffic accidents compared to females [9]. Moreover, evidence from Australia showed that men were more frequently involved in various crash types, including single-vehicle, high-speed, wet-condition, and nighttime crashes [28]. These findings collectively suggest that being female is generally associated with lower exposure to road traffic accidents across diverse contexts, reflecting differences in driving exposure, risk-taking behavior, and occupational engagement in traffic activities.

This study revealed that individuals with middle-income were positively associated with road traffic accidents compared to the rich. This aligns with findings from Organization for Economic Co-operation and Development (OECD) countries, where wealthier individuals, once reaching a certain income threshold, can afford safer vehicles, whereas lower-income growth often drives people toward riskier modes of transport such as motorcycles [29, 30]. However, this contrasts with findings from a study in Ethiopia, where being wealthy was positively associated with a higher occurrence of road traffic accidents, possibly due to increased vehicle ownership and greater road exposure among better-off households [9]. Similarly, in Uganda, wealthier rural residents face a steadily higher risk of injuries, suggesting increased exposure to accidents with rising income [31]. These variations highlight that the relationship between income and accident risk is context-dependent: in some low-income settings, initial increases in wealth may raise exposure before enabling access to safer transport. Consequently, road safety interventions should address both low-income groups and wealthier individuals prone to risky driving behaviors.

Our findings indicate that individuals reporting media exposure had 1.8 times greater odds of involvement in road traffic accidents (RTAs). However, this association should be interpreted cautiously. In this study, media exposure reflects general access to or engagement with media rather than media use while driving or riding. Media exposure may reflect underlying contextual factors such as urbanization, socioeconomic status, or increased mobility rather than a direct behavioral mechanism related to the crash event. Because the STEPS survey does not measure media use during travel or driving-related distractions, causality cannot be inferred. In addition, the cross-sectional design does not eliminate the possibility of reverse causality or residual confounding from unmeasured factors such as general risk-taking behavior or vehicle access [32]. Therefore, the observed association should be interpreted as a contextual correlation rather than evidence of media-related distraction. Media can also play a positive role in promoting road safety. For example, Rwanda’s “Gerayo Amahoro” campaign demonstrates that targeted public safety messaging can contribute to reducing RTA-related fatalities [15].

Place of residence significantly shapes the risk of road traffic accidents (RTAs) in Rwanda. Compared to residents of Kigali, individuals living in the Southern Province are less likely to experience RTAs, highlighting the influence of environmental and contextual factors beyond individual characteristics [33]. As Rwanda’s economic and administrative hub, Kigali experiences high traffic density, diverse road users, frequent congestion, and persistent accident hotspots, particularly along major arteries and downtown intersections, where speeding remains a leading contributor despite infrastructure improvements [34]. In contrast, the predominantly rural Southern Province has lower traffic volumes, simpler mobility patterns, and reduced inter-user conflicts, resulting in lower accident rates. These findings emphasize the need for context-specific interventions in Kigali, strategies such as intensified speed enforcement, traffic calming, and separation of pedestrians and cyclists are crucial, whereas in rural provinces, maintaining low exposure through highway speed control and targeted road improvements can help preserve protective effects.

Marital status was another significant factor. Compared to single individuals, those who were married or cohabiting and those who were divorced, separated, or widowed had 0.4 and 0.2 times lower odds of experiencing RTAs, respectively. In contrast, a study in Ethiopia reported that single drivers were less likely to be involved in traffic accidents compared to married drivers [35]. Similarly, findings from New Zealand suggest that unmarried individuals had a lower risk of fatal motor vehicle crashes compared to married individuals, possibly due to differences in driving behavior or exposure to traffic [36, 37]. In Rwanda, the protective effect among married or previously married adults may be linked to greater caution, higher adherence to traffic regulations, and lower involvement in high-risk travel modes such as motorcycles, which are more commonly used by younger, single adults.

Finally, this study found that helmet use, seatbelt use, and region of residence were not significantly associated with the occurrence of road traffic accidents (RTAs). The lack of association with place of residence aligns with findings from a Kenyan analysis of the 2020 Demographic and Health Survey but contrasts with evidence from Ethiopia, where urban residence was associated with more than twice the odds of RTAs compared with rural areas [8, 9]. The difference between Rwanda and Kenya versus Ethiopia may reflect variations in road network distribution, traffic density, and enforcement of road safety measures. In Rwanda, road safety interventions such as the Gerayo Amahoro campaign and nationwide Automated Speed Enforcement (ASE) cameras are implemented uniformly across regions, potentially reducing regional disparities in crash risk, whereas in Ethiopia, urban areas experience higher traffic congestion, vehicle density, and risk exposure, leading to greater RTA likelihood [15, 16].

Similarly, no significant association was observed between road traffic accidents and the use of safety devices, despite marked differences in reported compliance, characterized by high levels of consistent helmet use alongside relatively low and inconsistent seatbelt use. Compared with individuals who always used a seatbelt, those who reported occasional or no use did not have significantly different odds of experiencing an RTA, and a similar non-significant pattern was observed for helmet use. Although these findings may appear counterintuitive, they are consistent with evidence from Rwanda and other low- and middle-income countries showing that helmets and seatbelts primarily reduce injury severity and mortality rather than the likelihood of crash occurrence [38]. Reliance on self-reported safety behaviors may have introduced recall or social desirability bias, and including all road user categories may have diluted the effect of helmet use, which is primarily relevant for motorcyclists. Since this study focused on the occurrence of road traffic accidents rather than injury severity, the findings likely reflect that seatbelts and helmets are not associated with crash occurrence. Future research in Rwanda should focus on road traffic injuries to evaluate the effectiveness of safety devices in preventing injury and reducing crash-related harm.

### Strength & limitation

This study has several strengths. It used nationally representative, weighted data from the 2021–2022 Rwanda STEPS survey, enhancing the generalizability of the findings to adults aged 18–69 years. In addition, the analysis adjusted for multiple socio-demographic factors, allowing identification of independent associations with road traffic accidents (RTAs).

However, some limitations should be considered. The cross-sectional design does not allow causal inference between socio-demographic factors and RTAs. The use of self-reported data may introduce recall and social desirability bias, which could lead to under- or over-reporting of RTAs. In addition, 603 records (10.4%) were excluded due to incomplete RTA information, which may have introduced some selection bias if excluded participants differed from those included. Furthermore, as the STEPS survey primarily focuses on non-communicable disease risk factors, the injury module is limited. Important traffic-related variables such as the participant’s role in the crash (e.g., pedestrian, cyclist, or driver), driving exposure, and other contextual factors were not captured. The available variables on seatbelt and helmet use reflect general safety behaviors and are not linked to the reported accident.

Despite these limitations, the study provides useful baseline evidence on the prevalence of RTAs and their socio-demographic correlates in Rwanda and highlights the need for future studies with more detailed road safety data.

### Study implications

This study demonstrates that road traffic accidents (RTAs) in Rwanda are primarily driven by patterns of exposure, mobility, and socio-demographic characteristics, rather than by protective measures aimed at reducing injury severity. The elevated risk observed among males and middle-income individuals who are more frequently exposed to dense traffic and motorized transport highlights the need for targeted crash-prevention strategies focusing on economically active and highly mobile populations. These findings underscore that interventions should move beyond generic road safety messaging and adopt risk-based, evidence-driven approaches, in line with Rwanda’s National Road Safety Policy, which emphasizes reducing crash occurrence among high-risk groups.

The lower odds of RTAs among residents of the Southern Province compared to Kigali emphasize the role of urban traffic density, congestion, and mixed road use in shaping accident risk. Similarly, the protective effect of primary and secondary education suggests that risk perception, road safety knowledge, and adherence to traffic regulations are critical for preventing crashes. These findings support policy measures that integrate urban traffic management, road design improvements, and context-specific behavioral interventions, particularly in densely populated urban centers where exposure to traffic hazards is highest.

Importantly, the absence of significant associations between helmet or seatbelt use and accident occurrence confirms that these measures primarily reduce injury severity rather than crash risk. This distinction highlights the need for a dual strategy: combining upstream crash-prevention interventions such as traffic enforcement, driver education, and urban planning—with continued promotion of safety devices to mitigate harm when accidents occur. By identifying high-risk populations and contexts, this study provides evidence to guide data-driven interventions aligned with SDG 3.6, supporting Rwanda’s efforts to substantially reduce road traffic accidents and improve overall road safety outcomes. Future research should focus on the effectiveness of safety devices in preventing injury and mitigating crash-related harm, using prospective designs or linked police and health data to assess outcomes among specific road user groups, and exploring behavioral and environmental factors influencing consistent use of safety devices to guide targeted interventions.

## Conclusion

This study identified several socio-demographic factors associated with self-reported road traffic accidents in Rwanda. Lower odds of RTAs were observed among females, Southern Province, self-employed individuals, married or previously married participants, and those with primary or secondary education, whereas higher odds were observed among individuals from middle-income households and those reporting media exposure. These findings highlight the multifactorial nature of road traffic accident risk and indicate that RTA involvement varies across population subgroups. Given the cross-sectional design and reliance on self-reported data, the observed associations should not be interpreted as causal relationships. Nevertheless, the results provide evidence-informed insights that may help guide road safety policies and prevention strategies by identifying population groups that could benefit from targeted interventions. Future research using longitudinal designs and more detailed behavioral, environmental, and vehicle-related data such as driving exposure, alcohol consumption, speed, and road conditions is needed to better elucidate causal pathways and inform more effective and context-specific road safety interventions in Rwanda.

## Data Availability

The datasets used and analyzed during the current study are available from the corresponding author on reasonable request. The data supporting the findings were obtained from the 2021–2022 Rwanda STEPS Survey, which is accessible through formal permission from the Rwanda Biomedical Center (RBC).

## Abbreviations

ASE: Automated speed enforcement
CMHS: College of medicine and health sciences
DHS: Demographic and health survey
IRB: Institutional review board
NCD: Non-communicable disease
NISR: National institute of statistics of Rwanda
OECD: Organisation for economic co-operation and development
RBC: Rwanda biomedical center
RDHS: Rwanda demographic and health survey
RNP: Rwanda national police
RTA: Road Traffic Accident
RTI: Road traffic injury
STEPS: STEPwise approach to non-communicable disease risk factor surveillance
